# Hip Arthroscopy for Femoroacetabular Impingement With Initial Access to the Peripheral Compartment: A Systematic Review of Clinical Outcomes

**DOI:** 10.7759/cureus.76887

**Published:** 2025-01-03

**Authors:** Osamah Baig, Muzammil Akhtar, Ahmad Abulhasan, Muhammad Khattak, AbdulMuhaiman Khatib, Abdullah Khaja, Haider Syed

**Affiliations:** 1 Surgery, Lake Erie College of Osteopathic Medicine, Erie, USA; 2 Orthopedic Surgery, California Northstate University College of Medicine, Elk Grove, USA; 3 Orthopedic Surgery, Lake Erie College of Osteopathic Medicine, Erie, USA; 4 Physical Medicine and Rehabilitation, Lake Erie College of Osteopathic Medicine, Erie, USA; 5 Internal Medicine, Lake Erie College of Osteopathic Medicine, Erie, USA; 6 Anesthesiology, Lake Erie College of Osteopathic Medicine, Erie, USA; 7 Psychiatry, Lake Erie College of Osteopathic Medicine, Erie, USA

**Keywords:** central compartment, femoroacetabular impingement, hip arthroscopy, patient-reported outcome, peripheral compartment

## Abstract

The purpose of this review is to report clinical outcomes of patients undergoing hip arthroscopy for femoroacetabular impingement (FAI) with initial access to the peripheral compartment (PC) of the hip. A search following Preferred Reporting Items for Systematic Reviews and Meta-Analyses (PRISMA) guidelines was performed in the PubMed, Embase, and Scopus databases. Studies in which clinical outcomes were reported for patients who underwent hip arthroscopy for FAI with initial access to the PC were included. Data on study characteristics, patient demographics, radiographic outcomes, patient-reported outcomes (PROs), complications, and secondary surgery were extracted. Five studies with 881 patients and 976 hips (55.2% male, age range 32.1 to 48.4 years, follow-up range 3.0 to 74.4 months) were included. In two studies reporting radiographic measurements, the range of preoperative and postoperative alpha angle was 55.5° to 68.7° and 45.5° to 48.3°, respectively. The range of preoperative and postoperative lateral center-edge angles was 33.8° to 39.2° and 30.5° to 32.9°, respectively. Four studies reported one or more PRO measures with all demonstrating significant preoperative to postoperative improvement. The postoperative Non-Arthritic Hip Score (NAHS) and Visual Analog Score (VAS) pain scales ranged from 78.0 to 83.2 and 1.4 to 2.8, respectively. Complication rates ranged from 0.3% to 23.3%, with the rate of paresthesia specifically ranging from 0% to 8.1%. The rate of secondary surgery ranged from 0% to 6.3%. The rate of revision hip arthroscopy and conversion to total hip arthroplasty, specifically, ranged from 0% to 3.1% and 0% to 3.7%, respectively. This systematic review demonstrates that hip arthroscopy for FAI with initial access to the PC shows significant improvements in PROs with low rates of complications and secondary surgery.

## Introduction and background

Femoroacetabular impingement (FAI) is a clinical hip disorder that is characterized by abnormal and premature contact between the proximal femur and the acetabulum, leading to atypical joint biomechanics and damage to the articular cartilage and acetabular labrum. Patients typically present at a young age with motion-related or position-related groin pain that is most pronounced during hip flexion and internal rotation [1]. FAI can be further classified as having cam morphology, where the femoral head is nonspherical, or pincer morphology, where there is an overgrowth of the acetabulum relative to the femoral head. These can also occur in conjunction with one another [2]. With the increased risk of developing osteoarthritis [3] as well as other complications such as labral tears [4], FAI has become a focal point of exploration in understanding its pathophysiology, refining diagnostic approaches, and enhancing treatment methods.

In the surgical management of FAI, hip arthroscopy (HA) has emerged as a well-established procedure with an increase in incidence over the past decade [5]. Its popularity is supported by increases in symptomatic relief [6], patient-reported outcomes, and satisfaction [7], as well as a low incidence of postoperative complications [8]. The two principal surgical techniques that are used involve either a central compartment (CC) or peripheral compartment (PC) starting point. Central compartment procedures, although more frequently utilized, involve unique complications due to the necessity of traction [9]. Iatrogenic damage to the articular cartilage and acetabular labrum, as well as paresthesias not limited to pudendal nerve damage, are well-documented complications that can be avoided by reducing traction and surgical time [10].

Hip arthroscopy approached from the peripheral compartment, first described by Dorfmann et al. in 1999 [11], and later modified by Dienst et al. in 2001 [12], offers a useful alternative wherein the labrum and cartilage are spared [13]. The peripheral compartment is accessed without traction, resulting in a decreased risk of postoperative paresthesias [14]. The purpose of this systematic review is to report the clinical outcomes of patients undergoing hip arthroscopy with the peripheral compartment as the initial point of entry, highlighting the benefits and potential limitations of this technique.

## Review

Methods

Search Strategy

A search following Preferred Reporting Items for Systematic Reviews and Meta-analyses (PRISMA) guidelines was performed on December 1, 2024, in three databases: PubMed, Embase, and Scopus. The following boolean search phrase was used: (hip AND arthroscop* AND peripheral compartment). Studies were included if they performed hip arthroscopy for FAI and started the surgery with initial access to the PC. The exclusion criteria included case reports, review articles, technique articles, studies performed on animals, and expert opinions. 

Study Selection

Two independent reviewers reviewed studies according to the eligibility criteria from the initial database search. If they were not unanimous in their decision, a third reviewer was consulted to determine study inclusion or exclusion. All included articles underwent a rigorous search of their reference lists to determine whether additional studies fit our inclusion criteria and could be added to the systematic review.

Data Extraction

Study variables extracted from the studies included authors, study period, publication year, study design, patients, age, follow-up, surgical technique, type of FAI, procedures performed, preoperative and postoperative radiographic parameters, preoperative and postoperative patient-reported outcomes (PROs), complications, and incidence of secondary surgery. All extracted data were compiled for analysis using Microsoft Word (Microsoft Office 2011; Microsoft, Redmond, USA).

Quality Assessment and Risk of Bias

Methodological quality for all studies was assessed using the methodological index for non-randomized studies (MINORS) score, which is reported on a scale of 16 for non-comparative studies and 24 for comparative studies. Two authors scored each article in the systematic review. Each author scored the article individually before the authors reviewed their scores, and any discrepancies were resolved by re-reviewing the articles until a unanimous consensus was met. 

Statistical Analysis

Descriptive statistics such as means, percentages, standard deviations, ranges, medians, and interquartile ranges are reported in this review when applicable and when provided by the individual studies. P-values are also reported when available. A P < 0.05 was considered statistically significant. 

Results

Article Selection Process

**Figure 1:**
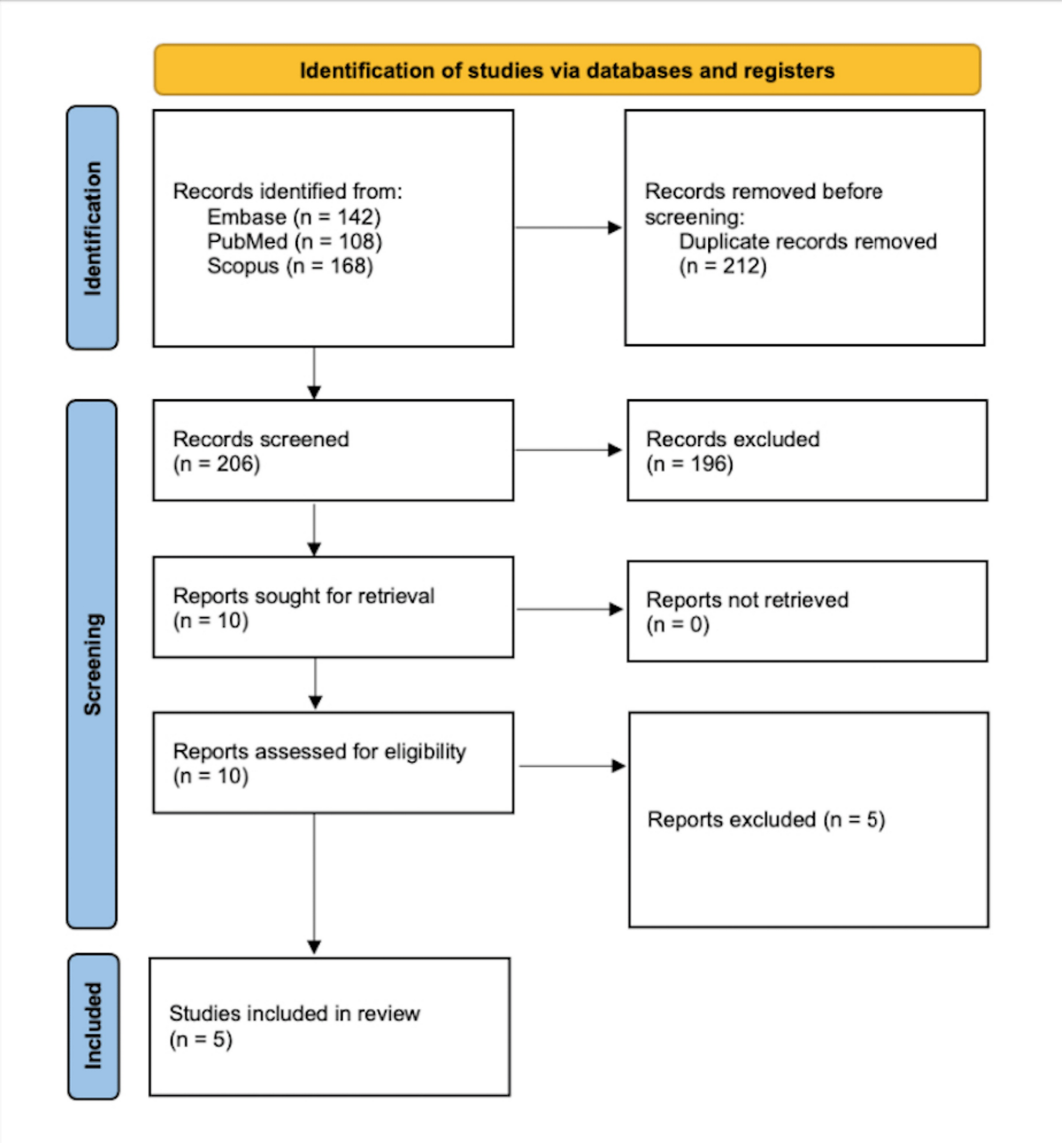
Flow chart depicting article selection process Preferred Reporting Items for Systematic Reviews and Meta-analyses (PRISMA) flow diagram for literature review to include desired studies.

The initial search in the PubMed, Embase, and Scopus databases yielded a total of 418 studies, of which 212 duplicates were removed. The titles and abstracts of the remaining 206 studies were screened against our inclusion and exclusion criteria, and of those, 196 studies were deemed irrelevant. The remaining 10 studies underwent full-text review, and of those, five studies were excluded. Five studies evaluating clinical outcomes of patients undergoing hip arthroscopy with initial access to the PC were included in this systematic review (Figure 1) [9, 13-16]].

Study Characteristics and Patient Demographics

The five studies were published between 2010 to 2024. Four studies were prospective case series that evaluated outcomes of only PC first hip arthroscopy, with a MINORS score ranging from 10 to 12. One study was a prospective cohort study that compared outcomes of PC versus CC first hip arthroscopy, with a MINORS score of 20. In total, there were 881 patients with 976 hips (55.2% male), with mean ages ranging from 32.1 to 48.4 years old. The mean follow-up time ranged from 3.0 to 74.4 months postoperatively. The type of FAI was reported in four studies, with 54.5% to 100.0% having cam deformities, 0% to 19.6% having pincer deformities, and 0% to 41.3% having mixed cam and pincer deformities. The specific procedures performed were reported in five studies, with the most common procedures being femoroplasty, acetabuloplasty, and labral repair (Table 1).

**Table 1:** Study Characteristics and Patient Demographics Age and Follow-up are reported as mean ± standard deviation (range) when possible. FAI, femoroacetabular impingement; NR, not reported; MINORS, methodological index for non-randomized studies

Author	Study Design	MINORS Score	Patients/Hips (M/F)	Age (years)	Follow-up (months)	FAI Morphology	Procedures Performed
Dantas et al. [15]	Prospective Case Series	12/16	154/160 (70/84)	36.0 ± 9.5 (16-59)	44.9 ± 11.0 (25-67)	Cam: 57.5% Pincer: 0.6% Mixed: 41.3%	Femoroplasty (99.4%), acetabuloplasty (45%), labral repair (76.8%), iliopsoas fractional lengthening (76.8%), subspine decompression (1.9%), removal of loose bodies (1.3%), acetabular microfracture (3.8%)
Özbek et al. [9]	Prospective Case Series	10/16	34/34 (14/20)	32.3 ± 12.5	24.0	NR	All underwent femoroplasty, acetabuloplasty, and labral repair
Wagner et al. [13]	Prospective Case Series	12/16	615/704 (428/276)	32.1 ± 9.2	74.4 ± 25.2 (12-108)	Cam: 54.5% Pincer: 19.6% Mixed: 25.9%	Combined femoroplasty and labral repair (46.7%), isolated femoroplasty (43.0%), isolated labral repair (5.2%)
Rupp and Duggan [14]	Prospective Cohort	20/24	30/30 (13/17)	48.4 (20-72)	3.0	Cam: 60.0% Pincer: 3.3% Mixed: 36.7%	Femoroplasty (100.0%), acetabuloplasty (56.7%), labral repair (50.0%), labral debridement (50.0%)
Dienst et al. [16]	Prospective Case Series	10/16	48/48 (25/33)	37.0 (17-65)	18.0	Cam: 100.0% Pincer: 0% Mixed: 0%	NR

Radiographic Parameters

Preoperative and/or postoperative radiographic parameters were reported in two studies. The range of the mean preoperative and postoperative alpha angle was 55.5° to 68.7° and 45.5° to 48.3°, respectively. The range of the mean preoperative and postoperative lateral center-edge angle (LCEA) was 33.8° to 39.2° and 30.5° to 32.9°, respectively. Additional reported radiographic parameters included the Tönnis angle, central column diaphyseal angle, and the presence of a crossover sign (Table 2).

**Table 2:** Preoperative and Postoperative Radiographic Parameters Values are reported as mean ± standard deviation. NR, not reported

Author	Measurement	Preoperative	Postoperative
Dantas et al. [15]	Central Column Diaphyseal Angle	131.7° ± 5.2°	NR
	Tönnis Angle	5.8° ± 4.2°	NR
	Presence of Crossover Sign (Yes)	23.8%	NR
	Alpha Angle	68.7° ± 10.0°	45.5° ± 4.4°
	Lateral Center-Edge Angle	33.8° ± 5.4°	30.5° ± 3.9°
Özbek et al. [9]	Alpha Angle	55.5° ± 2.9°	48.3° ± 2.6°
	Lateral Center-Edge Angle	39.2° ± 3.0°	32.9° ± 2.6°

Dantas et al. [15] reported the mean correction of the alpha angle was 23.1° (range, 5.9° to 46.7°), with the correction being significantly larger in male versus female patients (26.6° versus 20.6°, P < 0.01). The mean correction of the LCEA was 6.5° (range, -1.4° to 20.8°). Overall, the preoperative to postoperative alpha angle and LCEA values significantly improved (P < 0.01).

Patient-Reported Outcomes

Four studies reported one or more PRO measures. For studies reporting both preoperative and postoperative PROs, all PROs across all studies demonstrated significant improvement. The range of the mean preoperative and postoperative Non-Arthritic Hip Scores (NAHS) was 56.1 to 57.0 and 78.0 to 83.2, respectively. The range of the mean preoperative and postoperative Visual Analog Scale (VAS) Pain scores were 6.6 to 6.7 and 1.4 to 2.8, respectively (Table 3).

**Table 3:** Patient-Reported Outcomes, Complications, and Incidence of Further Surgery Values are reported as mean ± standard deviation. HOS-ADL, Hip Outcome Score–Activities of Daily Living; HOS-SSS, Hip Outcome Score–Sport-Specific Subscale; iHOT-12, International Hip Outcome Tool–12; LFCN, Lateral Femoral Cutaneous Nerve; mHHS, Modified Harris Hip Score; NAHS, Non-Arthritic Hip Score; NR, not reported; VAS Pain, Visual Analog Scale for Pain; WOMAC, Western Ontario and McMaster Universities Osteoarthritis Index; THA, total hip arthroplasty

Author	Patient-Reported Outcomes				Complications (N, %)	Secondary Surgery (N, %)
	Measurement	Preoperative	Postoperative	P-value		
Dantas et al. [15]	NAHS	56.1 (16-96)	83.2 (44-100)	< 0.001	Anterior dislocation at 3 months in firefighter who dislocated hip when taking down door with kick (1, 0.6%), heterotopic ossification (1, 0.6%), transient neurapraxia of pudendal nerve (1, 0.6%), transient mild symptoms related to LFCN due to location of anterior portal (12, 7.5%)	Revision hip arthroscopy (5, 3.1%), Conversion to THA (2, 1.3%)
Özbek et al. [9]	VAS Pain	6.7 ± 1.4	1.4 ± 1.0	< 0.05	NR	None
	mHHS	54.6 ± 5.1	87.2 ± 5.9	< 0.05		
	HOS-ADL	42.8 ± 9.5	88.4 ± 4.6	< 0.05		
	HOS-SSS	51.9 ± 7.9	76.2 ± 4.5	< 0.05		
Wagner et al. [13]	VAS Pain	6.6 ± 2.5	2.8 ± 2.3	< 0.05	Stress edema around femoral neck after femoroplasty which was evident on MRI, requiring percutaneous screw fixation of the femoral neck (2, 0.3%)	Revision hip arthroscopy (18, 2.6%), Conversion to THA (26, 3.7%)
	mHHS	NR	86.2 ± 13.1	-		
	iHOT-12	NR	78.7 ± 21.8	-		
Rupp and Duggan [14]	NR				Minor iatrogenic chondral injury (6, 20%), paresthesia (1, 3.3%)	NR
Dienst et al. [16]	NAHS	57.0 ± 19.0	78.0 ± 19.0	< 0.001	Permanent sensory disturbances of terminal branch of LFCN (2, 4.2%)	Surgical dislocation after 8 months to treat and initially underestimated acetabular retroversion (1, 2.1%), Conversion to THA (1, 2.1%)

Dantas et al. [15] reported that the mean improvement in the NAHS score was 27.7 points (range, -16 to 73), with female patients demonstrating a significantly greater improvement than male patients (31.5 versus 24.0, P < 0.016). They also found no association between the NAHS improvement and type of FAI, patient age, or presence of acetabular retroversion. Wagner et al. [13] reported that a positive response to an anchor question on whether patients would undergo surgery again or not was significantly correlated with satisfactory VAS Pain, International Hip Outcome Tool-12 (iHOT-12), and Modified Harris Hip Score (mHHS) scores (P < 0.001 for all).

Complications

Surgical complications or adverse events following surgery were reported in four studies, with complication rates ranging from 0.3% to 23.3%. The rate of nerve paresthesias specifically ranged from 0% to 8.1%. Iatrogenic chondral or labral injury ranged from 0% to 20.0%. Additional complications included hip dislocation, heterotopic ossification, and stress edema around the femoral neck following femoroplasty (Table 3).

Rupp and Duggan [14] compared iatrogenic injuries and complications following hip arthroscopy in patients with a PC versus CC starting point, with 30 hips in both groups. The iatrogenic injuries and complications for the PC versus CC groups are as follows: minor chondral injuries (20.0% versus 26.7%), moderate chondral injuries (0% versus 10.0%), severe chondral injuries (0% versus 0%), labral penetration (0% versus 6.7%), and paresthesias (3.3% versus 10.0%). The combined rate of chondral injuries and labral penetration was significantly higher in the CC compared to the PC group (43.4% versus 23.3%, P = 0.049). The rate of paresthesias was not significantly different between the two groups (P = 0.31).

Secondary Surgery

The incidence of secondary surgery was reported in four studies. The overall rate of secondary surgery ranged from 0% to 6.3%. The revision hip arthroscopy rate ranged from 0% to 3.1%. The conversion to total hip arthroplasty (THA) rate ranged from 0% to 3.7%. Additional secondary surgeries included pelvic osteotomy and surgical dislocation for the management of acetabular retroversion (Table 3).

Wagner et al. [13] specifically reported that in their cohort, the mean time to revision hip arthroscopy and conversion to total hip arthroplasty was 1.2 ± 2.1 years and 1.8 ± 1.2 years, respectively.

Discussion

In this systematic review, we included five studies that evaluated the clinical outcomes of 976 hips undergoing hip arthroscopy for FAI with initial access to the PC rather than the more traditional approach of initial access to the CC. The most important findings were that 1) there were significant preoperative to latest follow-up improvements in all PROs, 2) complication rates ranged from 0.3% to 23.3% with the rate of paresthesias specifically ranging from 0% to 8.1%, and 3) rates of secondary surgery ranged from 0% to 6.3% with the rates of revision hip arthroscopy and conversion to THA specifically ranging from 0% to 3.1% and 0% to 3.7% respectively. Overall, the initial access to the PC technique for hip arthroscopy demonstrated favorable PROs with low rates of complications and secondary surgery.

The initial access to the CC technique is the more common technique in hip arthroscopy with a couple of decades of evidence supporting its safety and reproducibility [17-18]. However, a cited concern with initial access to the CC is the risk of iatrogenic chondral or labral damage as accessing the CC is done only under fluoroscopic guidance and the surgeon’s feeling of the needle [19-20]. This risk of iatrogenic damage is particularly increased in cases with a tight hip joint such as the presence of coxa profunda, a hypertrophic labrum, or a limited ability to apply sufficient traction, all of which make initial access to the CC difficult [14, 21]. Therefore by initially accessing the PC, surgeons can perform progressive capsular release, rim resection, and labral detachment from the peripheral side. Additionally, the only major structure at risk of iatrogenic damage when accessing the PC is the anterolateral femoral head cartilage which can be avoided via fluoroscopic guided placement of the anterolateral portal. Subsequent access to the CC is then more feasible, especially in tight hip joints, and can be achieved under direct arthroscopic visualization, thus limiting the risk of iatrogenic damage [15, 19]. 

In the present systematic review, one study directly compared initial access to the PC versus the CC and found that the PC first group had a significantly lower rate of iatrogenic damage to cartilage and the labrum, whereas the rate of paresthesias was similar for both groups. Additionally, the total traction time was significantly lower in the PC compared to the CC start group (46 ± 26 minutes versus 73 ± 23 minutes, P = 0.002). The shorter traction time in the PC group was attributed to capsulotomy and labral exposure being first performed without traction. In the CC start groups, however, capsulotomy and labral exposure were performed under traction. The senior author of the study also reported that when starting with the PC, hip distraction was easier with less required traction force, however, a tensiometerwas not used to confirm this. Although traction times were significantly shorter in the PC group, the rate of nerve paresthesias was statistically similar, yet slightly less in the PC group (3.3% versus 10.0%, P = 0.31) [14].

Another concern is that making periportal or interportal capsulotomies when initially accessing the CC results in decreased tension of the iliofemoral ligament and drooping of the capsule onto the femoral head and neck which subsequently result in limited visibility when accessing the PC and may therefore require an extension of the capsulotomies [19]. A systematic review reported a higher prevalence of post-hip arthroscopy instability in patients with an insufficient hip capsule, thus emphasizing the importance of capsular preservation [22]. The benefit of initially accessing the PC is that the portals are established with small periportal capsulotomies, which preserves fluid pressure, allows for ballooning of the capsule for adequate exposure of the PC, and provides maximal capsular preservation [9, 15, 23].

This systematic review must be considered in the context of its limitations. The data was derived from only five studies, all of which were level III or IV evidence. Only one study directly compared initial access to the PC versus CC whereas the rest of the studies were all case series of patients only undergoing PC first hip arthroscopy. Additionally, all five studies were performed at a single center by a single surgeon which may limit the generalizability of the conclusions. The follow-up times of the studies highly varied with the range of follow-up times being 3.0 to 74.0 months. 

## Conclusions

Hip arthroscopy for FAI with initial access to the PC demonstrated significant improvements in PROs and was associated with low rates of complications, including iatrogenic damage, nerve paresthesias, and secondary surgeries. Compared to the traditional approach of initial CC access, the PC technique reduced traction times and minimized the risk of damage to intra-articular structures, particularly in cases of tight hip joints. These findings highlight the efficacy and safety of this approach while emphasizing its potential to improve surgical outcomes and patient satisfaction. However, further studies with long-term follow-up are needed to validate these results and assess broader applicability.
